# Resource Quantity and Quality Determine the Inter-Specific Associations between Ecosystem Engineers and Resource Users in a Cavity-Nest Web

**DOI:** 10.1371/journal.pone.0074694

**Published:** 2013-09-11

**Authors:** Hugo Robles, Kathy Martin

**Affiliations:** 1 Department of Forest and Conservation Sciences, Centre for Applied Conservation Research, University of British Columbia, Vancouver, British Columbia, Canada; 2 Pacific Wildlife Research Centre, Environment Canada, Delta, British Columbia, Canada; University of Manitoba, Canada

## Abstract

While ecosystem engineering is a widespread structural force of ecological communities, the mechanisms underlying the inter-specific associations between ecosystem engineers and resource users are poorly understood. A proper knowledge of these mechanisms is, however, essential to understand how communities are structured. Previous studies suggest that increasing the quantity of resources provided by ecosystem engineers enhances populations of resource users. In a long-term study (1995-2011), we show that the quality of the resources (i.e. tree cavities) provided by ecosystem engineers is also a key feature that explains the inter-specific associations in a tree cavity-nest web. Red-naped sapsuckers ( *Sphyrapicus* *nuchalis* ) provided the most abundant cavities (52% of cavities, 0.49 cavities/ha). These cavities were less likely to be used than other cavity types by mountain bluebirds ( *Sialia* *currucoides* ), but provided numerous nest-sites (41% of nesting cavities) to tree swallows ( *Tachycineta* *bicolour* ). Swallows experienced low reproductive outputs in northern flicker ( *Colaptes* *auratus* ) cavities compared to those in sapsucker cavities (1.1 vs. 2.1 fledglings/nest), but the highly abundant flickers (33% of cavities, 0.25 cavities/ha) provided numerous suitable nest-sites for bluebirds (58%). The relative shortage of cavities supplied by hairy woodpeckers ( *Picoides* *villosus* ) and fungal/insect decay (<10% of cavities each, <0.09 cavities/ha) provided fewer breeding opportunities (<15% of nests), but represented high quality nest-sites for both bluebirds and swallows. Because both the quantity and quality of resources supplied by different ecosystem engineers may explain the amount of resources used by each resource user, conservation strategies may require different management actions to be implemented for the key ecosystem engineer of each resource user. We, therefore, urge the incorporation of both resource quantity and quality into models that assess community dynamics to improve conservation actions and our understanding of ecological communities based on ecosystem engineering.

## Introduction

Key ecosystem engineers are organisms that facilitate and modulate resource availability to other organisms via physical modification or creation of habitat [1,2]. Because ecosystem engineering is a phenomenon present in most ecological systems [3], strategies that focus on ecosystem engineering can provide new insights for conservation biology [2]. Indeed, numerous recent studies show the importance of ecosystem engineering in structuring ecological communities in a wide variety of systems [4–12]. However, despite the recognized importance of ecosystem engineering as a structural force that enhances the biodiversity of ecological communities, the underlying mechanisms of the inter-specific associations between ecosystem engineers and the users of the resources provided via ecosystem engineering are poorly known in most ecosystems. A better knowledge of these mechanisms is particularly relevant to elucidate if resources supplied by different ecosystem engineers provide different benefits to a given resource user [6]. If this proved true, niche differentiation associated with the differential quality of resources provided by ecosystem engineers might promote coexistence of ecologically similar species by overcoming potential negative effects of inter-specific competition [13,14]. Consequently, management actions oriented to preserve populations of resource users require proper identification of the respective roles of potential key ecosystem engineers for each resource user.

The poor knowledge of the mechanisms underlying the specific associations between ecosystem engineers and resource users is partly due to the fact that the outcome of ecosystem engineering has often been assessed by examining experimental or correlative relationships between the presence or abundance of ecosystem engineers (or the resources they provide) and the presence, abundance, species richness or species diversity of resource users. This study approach assumes that an increase in the abundance of resource users (or their species richness or diversity) in response to the increase in resource quantity indicates a positive effect of ecosystem engineers on populations or communities. However, the increase of a particular ecosystem engineer may increase the abundance or species richness and diversity of resource users in the short-term, but the long-term impact of this action might be limited or even negative if the reproductive output of resource users is reduced considerably when using that particular resource. In addition, most previous studies have assessed single species of resource users and/or single species of ecosystem engineers, but different species of ecosystem engineers are expected to provide resources that differ in quality for each particular resource user [6]. A study approach that can improve our understanding of the mechanisms underlying the specific associations between ecosystem engineers and resource users is, therefore, to investigate resource use decisions and changes in the reproductive output of several resource users (i.e. the variation in resource quality) in relation to the resource types provided by multiple ecosystem engineers. Such a study approach would allow us to assess whether resource quality for each resource user differs among the resources provided by different ecosystem engineers.

In this paper, we investigated the mechanisms underlying the inter-specific associations between ecosystem engineers and resource users in a tree cavity-nesting bird community. Cavity-nesting birds, which depend on tree-cavities for nesting in forest ecosystems (e.g. [15]), are hierarchically structured in nest web communities according to their mode of acquiring cavities (e.g. [16,17]). Primary cavity nesters are able to build their own breeding cavities through excavation (e.g. [16]), whereas secondary cavity-nesting birds require cavities provided by avian excavators or by fungal/insect decay (e.g. [16,18]). Fungal and insect infections, which may be stimulated by abiotic factors such as fires, windstorms and snow damage [19], induce cavity formation either directly by progressive heartwood decay or indirectly by providing suitable substrate for woodpecker excavation (e.g. [20]). Thus, avian excavators and rot fungi/insects function as key ecosystem engineers that supply nest-sites to secondary cavity users [1].

We investigated the mechanisms underlying the specific associations between ecosystem engineers and resource users by examining the responses of two secondary cavity nesters (the mountain bluebird *Sialia* *currucoides* and the tree swallow *Tachycineta* *bicolor* ) to the variation in the quantity and quality of tree-cavities (i.e. the critical resources) supplied by four types of ecosystem engineers: three avian excavators (northern flicker *Colaptes* *auratus* , red-naped sapsucker *Sphyrapicus* *nuchalis* , hairy woodpecker *Picoides* *villosus* ) and rot fungi/insects [16]. First, we assessed the hypothesis that cavity quantity explains the inter-specific associations between ecosystem engineers and secondary cavity users by calculating the density of cavities provided by each ecosystem engineer and the number of these cavities that were used by each secondary cavity user. We expected that secondary cavity nesters used higher numbers of cavities supplied by the ecosystem engineers that provided the most abundant cavities. We then assessed the hypothesis that cavity quality explains the inter-specific associations between ecosystem engineers and secondary cavity nesters by assessing the probabilities of using cavities provided by different ecosystem engineers and the reproductive outputs of cavity users in those cavities. According to this alternative hypothesis, we expected that cavity quality is a species-specific attribute so that bluebirds and swallows had different probabilities of use and reproductive outputs in cavities supplied by different ecosystem engineers.

## Methods

### Ethics statement

The surveys were conducted primarily on public lands. The Department of National Defence allowed us to conduct field research on two sites and lease holders allowed us to work on three sites. All field activities were in agreement with federal and provincial legislation.

### Study area and species

We collected the data in 35 sites (7-32 ha each) in the Cariboo-Chilcotin region (52^°^08’30″ N, 122^°^08’30″ W) of interior British Columbia, Canada. The study included two forest types: continuous mixed forests (27 sites) and aspen groves (8 sites). Continuous forest sites were mature mixed deciduous-coniferous forests (80 to >120 years) composed of Lodgepole pine ( *Pinus* *contorta* var. *latifolia* , 42% of trees), Douglas-fir ( *Pseudotsuga* *menziesii* , 28%), hybrid white spruce ( *Picea* *glauca* *x engelmannii*, 18%) and trembling aspen ( *Populus* *tremuloides* , 12%). Black cottonwood ( *Populus* *balsamifera* ), alder ( *Alnus* spp.), paper birch ( *Betula* *papyrifera* ), and willow ( *Salix* spp.) were present in very low proportions [21]. The continuity of continuous mixed forests was occasionally disrupted by small grasslands, shallow ponds and selective harvesting. Aspen groves were principally composed of trembling aspen (54%) and, to a lesser extent, of lodgepole pine (38%) and Douglas-fir (8%). Aspen grove sites consisted of a few small groves (0.2-5 ha) surrounded by an extensive matrix of grasslands and shallow ponds [21].

Mountain bluebirds (~30 g) and tree swallows (~21 g) are secondary cavity-nesting passerines that use existing cavities for nesting. Both cavity nesters are insectivores that require open areas for foraging [22,23]. Thus, the extensive matrix of grasslands and shallow ponds surrounding aspen groves provides swallows and bluebirds with large areas of suitable foraging habitat. The availability of foraging habitat for swallows and bluebirds is limited in continuous forests, where these birds forage on the interspersed grasslands, shallow ponds and clear-cuts within the forest. Populations of bluebirds remain stable within their distribution range in North America [22], whereas swallow populations have declined in recent decades particularly in the areas of greatest swallow density within its distribution range [23]. Populations of both secondary cavity users have increased during last years in our study area [24].

### Reproductive parameters, cavity densities and cavity characteristics

We searched for nests of cavity excavators and secondary cavity users within the sites between May and July 1995-2011 (range of 6 to 13 years of data per site, mean ± SE = 10.8 ± 0.5 years). We conducted nest surveys for an average of 6-7 observer-hours per sampling site per week [16]. We found nest cavities by observing behavior of adults and listening for begging nestlings. We inspected cavity contents by either using a ladder, flashlights and mirrors, or with a video camera system mounted on a pole (TreeTop Peeper; Sandpiper Technologies, Manteca, CA). We found most nests during the laying or early incubation stage. Tree cavities with at least one egg or nestling were considered active nests, which were visited every 4-7 days to monitor reproduction. We recorded clutch size and the number of large nestlings (i.e. pre-fledglings). We confirmed clutch size when we observed the same number of eggs in two visits to a given nest with no indication of nest predation or abandonment during the laying period. To obtain an accurate estimation of fledgling number, we counted the number of large nestlings within the last 5 days before fledging minus the number of large nestlings left dead in the cavity after fledging, if any. We considered nests as successful if we observed fledglings or large nestlings about to fledge. For unsuccessful nests, we tried to identify cause of failure by visual inspections of the nesting cavities.

From 1999, we systematically monitored the cavities we found during routine searches in previous years until they were no longer available for nesting (details in [21]). These methods allowed us to know the history of each cavity, which is necessary to estimate the density of cavities provided by each ecosystem engineer and available to bluebirds and swallows across years. Because we cannot exclude the possibility that a small number of cavities may have been missed during surveys, our estimations provide minimum densities of cavities potentially available for nesting. However, we performed, to our knowledge, the most exhaustive surveys of cavities in a cavity-nesting bird study, and we believe that our estimate of cavity densities is a reliable estimate of cavity abundances on our study plots. We excluded data from 1995 to 1998 because the cavity formation agent (ecosystem engineer) of some cavities at the beginning of the study was unknown. Hence, we calculated cavity density supplied by ecosystem engineers from 1999 to 2011 during which cavity history, including the identity of the ecosystem engineer that formed the cavity, was known. In addition, we used the data from 1999 to 2011 in analyses that assessed the probability of cavity use by bluebirds and swallows (see below), but we used the whole data set (1995-2011) to assess the reproductive parameters of secondary cavity users and the characteristics of the cavities supplied by ecosystem engineers (see below). The number of years used for each analysis is indicated in the results section.

When the breeding season of each year was over, we measured the distance to forest edge and cavity height above the ground level for all the cavities available (used and unused in a given year) to bluebirds and swallows. In a subset of cavities that were accessible by a ladder (i.e. up to 5.2 m high on stable trees), we also recorded the internal width of the cavities as the horizontal distance from the inside edge of the lower cavity lip to the back of the cavity. Finally, we measured the depth of accessible cavities from the lower lip of the cavity entrance to the bottom of the cavity [24].

### Statistical analyses

#### Density of cavities supplied by ecosystem engineers

We used a generalized linear mixed model (GLMM) with a Gaussian error distribution and an identity link function to assess potential differences in the cavity density (cavities ha^-1^) supplied by the ecosystem engineers. Ecosystem engineer identity was fitted as a fixed term. Year and site identity were fitted as random terms to control for multiple observations within the same years and sites.

#### Characteristics of cavities supplied by ecosystem engineers

We also used Gaussian GLMMs to compare cavity entrance size, cavity volume, cavity height and distance to forest edges among the cavities supplied by the ecosystem engineers. If cavities had entrances with irregular or oval shapes, we took the narrowest dimension as a measure of cavity entrance size, as smaller entrances may reduce predation risk or prevent access to larger birds [18,25]. We assumed that cavities were cylindrical and used internal cavity width and depth to calculate cavity volume as follows: V=πw^2^d, where w is cavity width and d is cavity depth [24]. Year, site, and cavity identity nested within site identity, were fitted as random terms to control for multiple observations within the same years, cavities and sites.

#### Cavity use of secondary cavity users

We used GLMMs with binomial error distributions and logit link functions to assess cavity use of swallows and bluebirds. The binomial dependent variable was cavity use (used for nesting in a given year=1, non-used = 0). In addition to ecosystem engineer identity, the distance from cavities to forest edges and habitat type (continuous mixed forest vs. aspen groves) were fitted as fixed terms. This modeling approach allowed us to examine the influence of cavity type (i.e. ecosystem engineer identity) while accounting for other habitat attributes that may influence cavity use. Year, site, and cavity identity nested within site identity, were fitted as random terms.

We used an information-theoretic approach [26] to assess which characteristics better depicted cavity use by swallows and bluebirds. For each species, we ran a set of 8 models containing combinations of the specific variables as well as the intercept-only model (i.e. the null model). We ranked these models according to Akaike’s Information Criterion corrected for small sample sizes (AICc) and Akaike model weights [26,27]. Models with low AICc values are considered to be well supported by the data [26]. Akaike model weights quantify the support of every model by the data, where higher weights indicate better explanatory power. The sum of all model weights is 1 [26]. When the null model was not classified as the highest-ranked model, we assessed variable influence by calculating model-averaged parameter estimates and their associated standard errors across the set of all candidate models.

#### Reproductive parameters of secondary cavity users

We used binomial GLMMs to assess nesting success (success=1, failure=0). Ecosystem engineer identity was fitted as a fixed term. We also included the distance from cavities to forest edges, habitat type, cavity entrance size, cavity volume and cavity height above the ground level as fixed terms, as these factors may influence nesting success of cavity nesters [25,28,29]. In addition, we included the stage at which nests were found (pre-laying vs. egg laying and incubation vs. nest with nestlings) as fixed terms to control for its potential influence on the variation of nesting success [30]. Given that we expected that the potential influence of cavity type (i.e. ecosystem engineer identity) on nesting success may be strongly correlated with the variation in cavity dimensions among ecosystem engineers, ecosystem engineer identity and cavity dimension variables (cavity entrance size, volume and height) were not fitted in the same models. Thus, we ran two different sets of models. The first model set included combinations of distance to edges, habitat type and ecosystem engineer identity as explanatory variables. In the second model set we fitted cavity dimension variables instead of ecosystem engineer identity as fixed terms while controlling for distance to edge and habitat type.

We used Poisson GLMMs to examine clutch size and the number of fledglings (successful broods only). One exception was clutch size of bluebirds, which we analyzed using Gaussian GLMMs because Gaussian error distributions improved model fit. In addition to ecosystem engineer identity, we fitted other variables that may influence clutch size and fledgling number as fixed terms: the distance from cavities to forest edges, habitat type and cavity volume. Similar to analyses of nesting success, we ran two different sets of models that contained combinations of different variables; one model set with ecosystem engineer identity and the second one with cavity volume as fixed terms while controlling for habitat type and distance to edges in both model sets.

In all models of reproductive parameters we fitted year, site, and cavity identity nested within site identity as random terms. Model and variable selection followed the same methods as those indicated for analyses of cavity use. We conducted all statistical analyses in this paper using R 2.14.1 [31].

## Results

### Abundance of cavities supplied by ecosystem engineers

**Figure 1:**
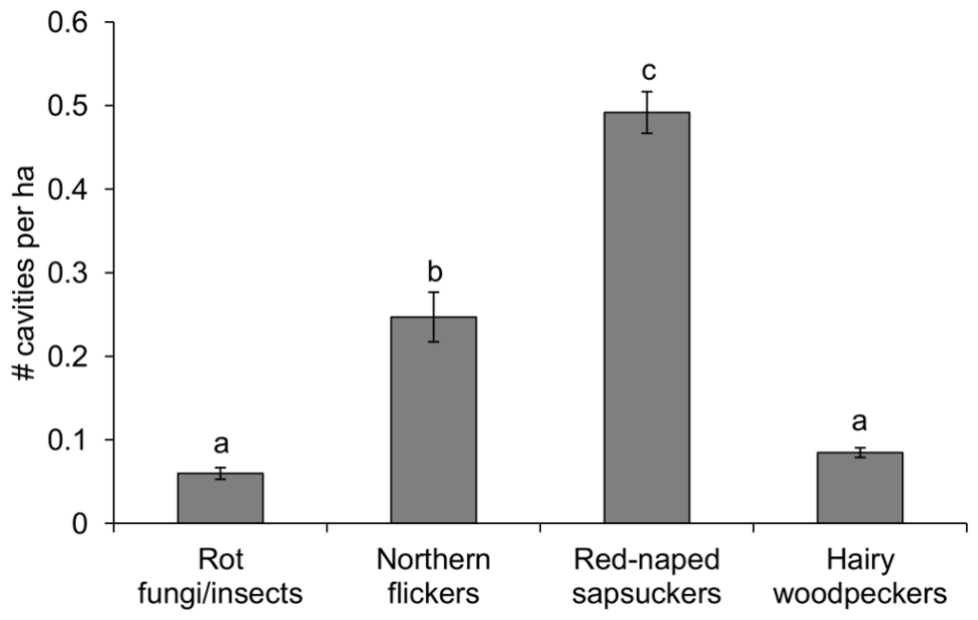
Average density of cavities provided by the ecosystem engineers in a tree cavity nesting community. Different letters above error bars (i.e. ±SE) indicate significant differences (*p*<0.001) among groups (Tukey’s post-hoc test).

We calculated the density of cavities supplied by each ecosystem engineer for a total of 315 site-years from 1999 to 2011. Sapsuckers provided the highest average cavity density, whereas the density of cavities supplied by hairy woodpeckers and fungal-insect decay were significantly lower than those of sapsuckers and flickers (Figure 1). Overall, sapsuckers provided 52% of cavities (n=2287 cavity-years) available for secondary cavity nesters, followed by flickers (33%, n=1453), hairy woodpeckers (9%, n=409) and fungal-insect decay (6%, n=277).

### Characteristics of cavities supplied by ecosystem engineers

**Figure 2:**
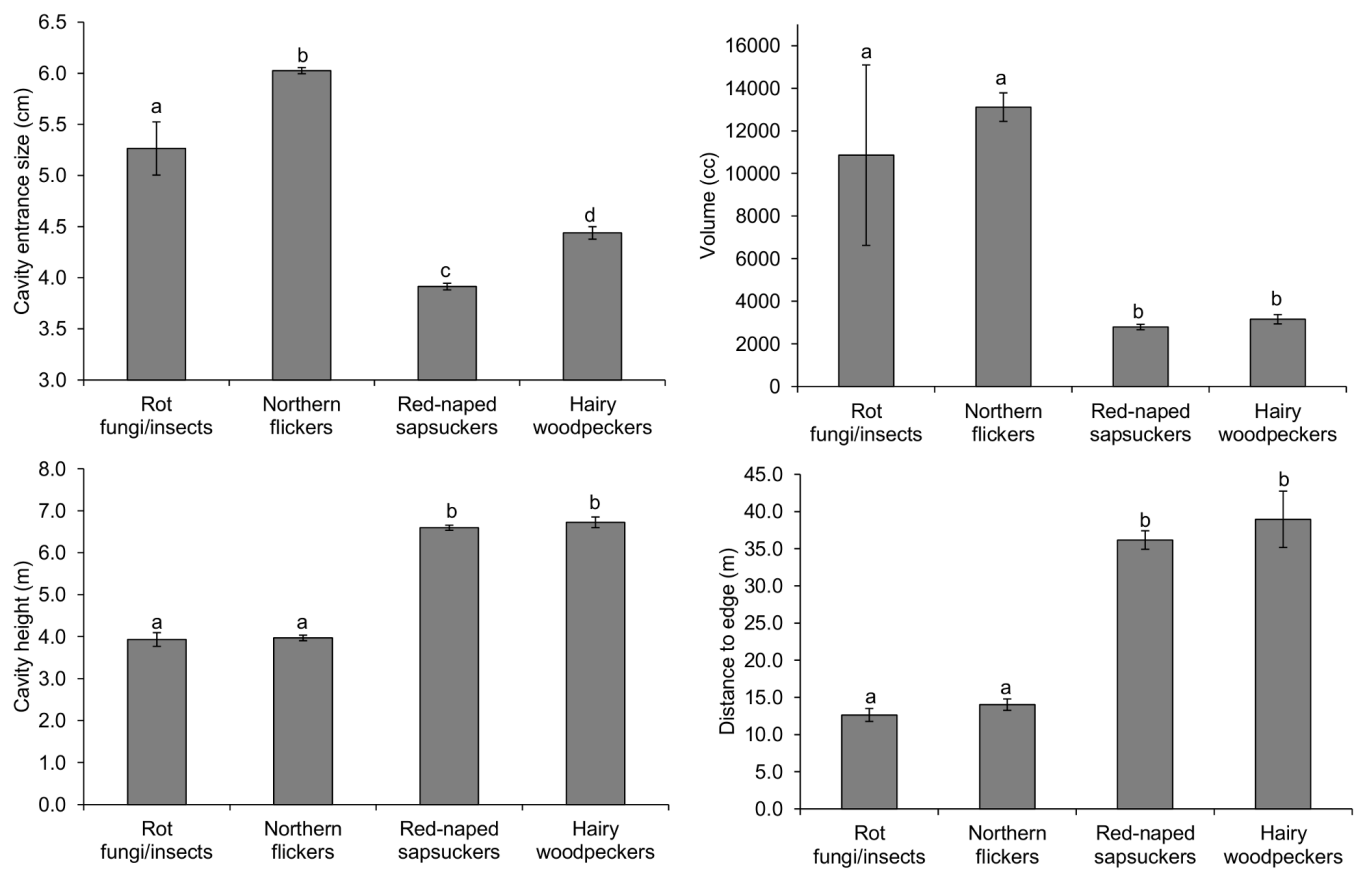
Mean entrance size, volume, height above the ground level and distance to forest edge of cavities. Different letters above error bars (i.e. ±SE) indicate significant differences among groups (Tukey’s post-hoc test: all *p*<0.001 except differences in cavity volume between cavities supplied by hairy woodpeckers and by fungal/insect decay for which *p*=0.055).

From 1995 to 2011, we measured the volume and the entrance size of 1345 and 1502 cavity-years, respectively, supplied by the four ecosystem engineers. Additionally, we calculated cavity height on the tree and the distances from cavities to forest edges in 5780 cavities. Flicker cavities had the largest entrances, followed by cavities supplied by fungal/insect decay, hairy woodpeckers and sapsuckers (Figure 2). The volume of cavities supplied by flickers and fungal/insect decay was significantly greater than that of hairy woodpecker and sapsucker cavities (Figure 2). Cavities provided by flickers and fungal/insect decay were located lower on the trees and closer to forest edges than sapsucker and hairy woodpecker cavities (Figure 2).

### Cavity use of secondary cavity users

Swallows used mostly sapsucker and flicker cavities for nesting (41.1% and 30.9% of 382 nests in sapsucker and flicker cavities, respectively), but they also used hairy woodpecker cavities (13.1%) and cavities supplied by fungal/insect decay (6.5%). Bluebirds mostly used flicker cavities as nest-sites (57.6% of 368 nests), with lower proportions of nests in cavities supplied by fungal/insect decay (14.7%), hairy woodpeckers (13.3%) and sapsuckers (12.0%).

**Figure 3:**
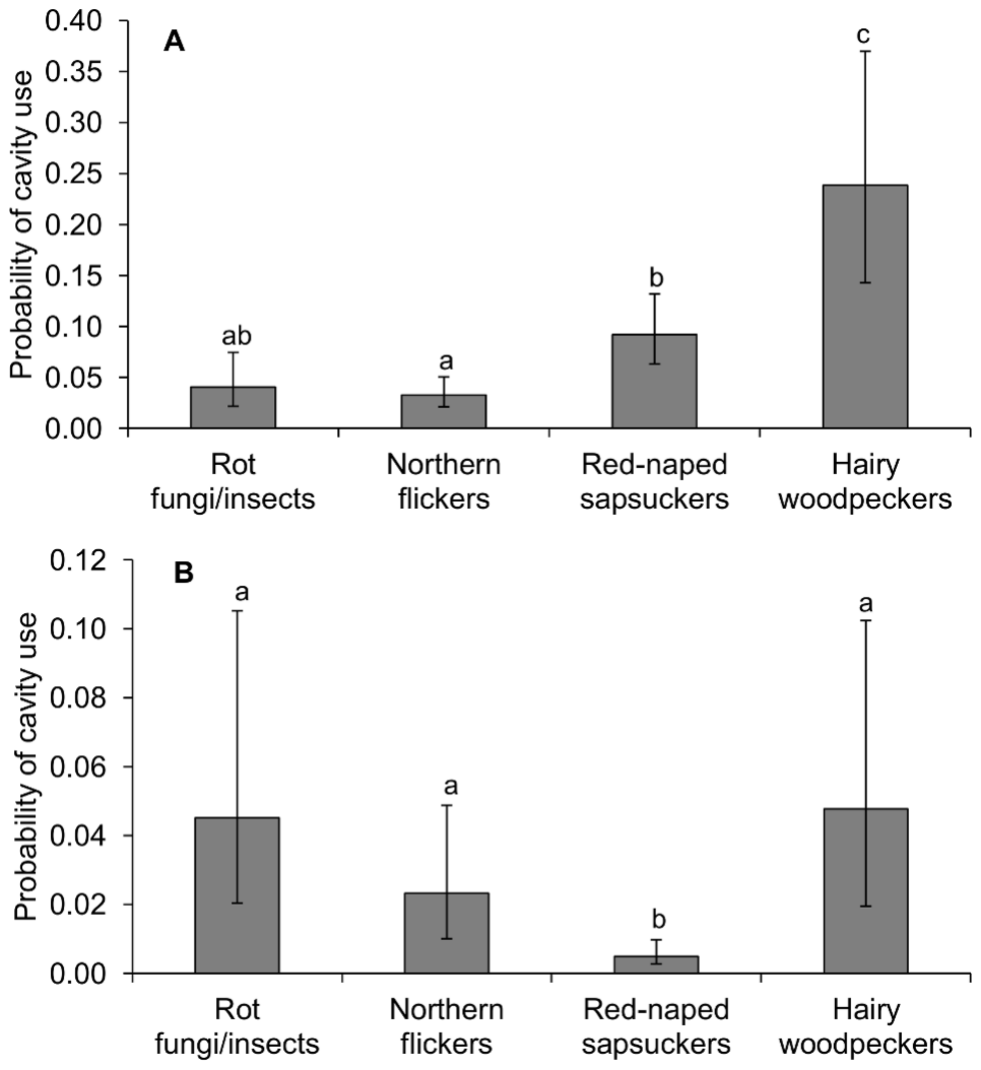
Probability of use by swallows (A) and bluebirds (B) of cavities provided by the ecosystem engineers. The probabilities of cavity use are the predicted probabilities calculated from model-averaged parameter estimates across the set of all candidate models that depicted cavity use (see tables 1 and 2). Different letters above error bars (i.e. ±SE) indicate significant (p<0.05) differences among groups according to Tukey’s post-hoc test for the best models in table 1.

We compared the characteristics of cavities used by swallows (n=330 cavity-years, 1999 to 2011) and bluebirds (n=306) with those of unused cavities (n=3438). For swallows, model selection of analyses of nest site use yielded one high ranked model that accounted for most weight in the candidate model set (Table 1). This model contained ecosystem engineer identity, habitat type and distance to forest edge as predictors of cavity use. Swallows used cavities close to forest edges (mean distance to edge ± SE: used vs. non-used cavities = 10.8 ± 0.8 m vs. 31.8 ± 1.1 m, Table 2). Cavities in aspen groves were more likely to be used by swallows than cavities in continuous forests (habitat type: proportion of cavities used in aspen groves vs. continuous forest = 0.154 vs. 0.046, Table 2). Flicker cavities had a lower probability of being used than sapsucker and hairy woodpecker cavities (Figure 3). Swallows were more likely to use hairy woodpecker cavities than the other cavity types, whereas the probability of using cavities supplied by fungal/insect decay did not differ significantly from that of cavities supplied by flickers and sapsuckers (Figure 3).

**Table 1:** Model selection of analyses that examined tree cavity use by swallows and bluebirds.

Species	Models	df	LogLik	AICc	ΔAICc	Weight
**Swallow**	Ecosystem engineer, habitat, edge	9	-901.38	1820.81	0.00	0.95
**Swallow**	Habitat, edge	6	-908.08	1828.18	7.37	0.02
**Swallow**	Ecosystem engineer, habitat	8	-906.29	1828.62	7.81	0.02
**Swallow**	Ecosystem engineer, edge	8	-907.41	1830.86	10.05	0.01
**Swallow**	Habitat	5	-913.96	1837.93	17.12	0.00
**Swallow**	Edge	5	-920.98	1851.98	31.17	0.00
**Swallow**	Ecosystem engineer	7	-919.01	1852.05	31.24	0.00
**Swallow**	Intercept-only model	4	-931.69	1871.39	50.58	0.00
**Bluebird**	Ecosystem engineer, habitat, edge	9	-757.49	1533.03	0.00	0.93
**Bluebird**	Ecosystem engineer, habitat	8	-761.06	1538.17	5.14	0.07
**Bluebird**	Ecosystem engineer, edge	8	-764.64	1545.32	12.29	0.00
**Bluebird**	Ecosystem engineer	7	-770.91	1555.84	22.82	0.00
**Bluebird**	Habitat	5	-775.32	1560.66	27.63	0.00
**Bluebird**	Intercept-only model	4	-784.49	1576.99	43.97	0.00
**Bluebird**	Edge	5	-2674.87	5359.75	3826.73	0.00
**Bluebird**	Habitat, edge	6	-7256.51	14525.04	12992.01	0.00

The characteristics of used cavities (distance from cavities to forest edge, ecosystem engineer identity and habitat type [aspen groves vs. continuous forest]) were compared to those empty cavity-year events. AICc: AIC corrected for small sample size, ΔAICc: difference in AICc to the best model. Models of the model set for each bird species are ranked according to their Akaike weight (Weight).

**Table 2:** Model-averaged parameter estimates and standard errors for analyses that examined tree cavity use of swallows and bluebirds.

Species	Parameters	Estimate ± SE	*z*	*p*
**Swallow**	Intercept	-1.161 ± 0.629	1.846	0.065
**Swallow**	Ecosystem engineer (rot fungi/insects)	-2.003 ± 0.645	3.106	**0.002**
**Swallow**	Ecosystem engineer (flickers)	-2.226 ± 0.453	4.910	**<0.001**
**Swallow**	Ecosystem engineer (sapsuckers)	-1.128 ± 0.406	2.778	**0.005**
**Swallow**	Distance to edge	-0.023 ± 0.009	2.656	**0.008**
**Swallow**	Habitat (continuous forest)	-2.050 ± 0.474	4.325	**<0.001**
**Bluebird**	Intercept	-2.839 ± 0.747	3.802	**<0.001**
**Bluebird**	Ecosystem engineer (rot fungi/insects)	-0.327 ± 0.974	0.336	0.737
**Bluebird**	Ecosystem engineer (flickers)	-0.767 ± 0.703	1.092	0.275
**Bluebird**	Ecosystem engineer (sapsuckers)	-2.734 ± 0.830	3.294	**<0.001**
**Bluebird**	Distance to edge	-0.023 ± 0.020	1.167	0.243
**Bluebird**	Habitat (continuous forest)	-1.751 ± 0.556	3.150	**0.002**

Significant values (*p*≤0.05) are highlighted in bold. See footnotes in table 1 for description of parameters.

Model selection of cavity use for bluebirds also yielded one high ranked model that accounted for most weight in the candidate model set (Table 1). Bluebirds were more likely to use cavities in aspen groves than in continuous forests (habitat type: proportion of cavities used in aspen groves vs. continuous forest = 0.159 vs. 0.033, Table 2). In addition, bluebirds nested in cavities close to forest edges (mean distance to edges ± SE: used vs. non-used cavities = 10.0 ± 0.8 m vs. 31.8 ± 1.1 m), although this effect was not significant after controlling by year, site and cavity identity (Table 2). Sapsucker cavities were the least likely to be used compared to the remaining cavity types (Figure 3). Given that sapsuckers provide the smallest cavities to these secondary cavity nesters (see above and Figure 2), we hypothesized that bluebirds preferred sapsucker cavities with larger entrances for nesting. We addressed this hypothesis by using binomial GLMMs to examine bluebird use (used=1, non-used = 0) of sapsucker cavities with cavity entrance size and volume as fixed terms and cavity identity as a random term. Cavity volume did not differ between sapsucker cavities used and not used by bluebirds (parameter estimate ± SE = -0.00005 ± 0.00008, *z*=-0.603, *p*=0.55, n=354 sapsucker cavities), but sapsucker cavities used by bluebirds had larger entrances than those non-used (mean cavity entrance size ± SE: used vs. non-used = 4.3 ± 0.1 cm vs. 3.9 ± 0.0 cm, parameter estimate ± SE = 0.997 ± 0.239, *t*=4.172, *p*<0.001, n=397).

### Nesting success of secondary cavity users

**Figure 4:**
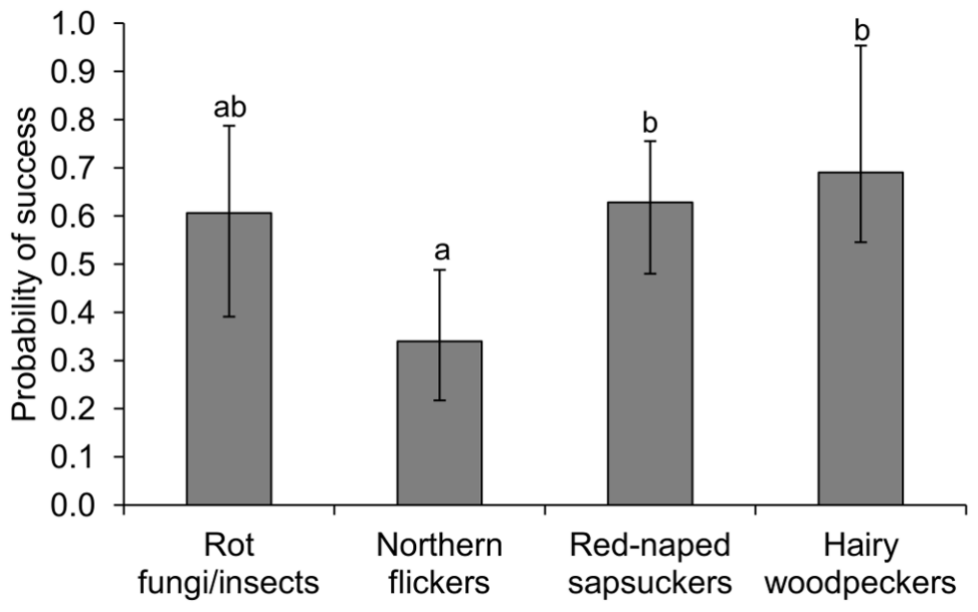
Probability of nesting success of swallows in cavities supplied by the ecosystem engineers. Nesting success probabilities are the predicted probabilities calculated from model-averaged parameter estimates across the set of all candidate models that depicted swallow nesting success (see tables 3 and 4). Different letters above error bars (i.e. ±SE) indicate significant differences among groups at p<0.1 (Tukey’s post-hoc test for the best model in Table 3; flicker vs. hairy woodpecker: p=0.068, flicker vs. sapsucker: *p*=0.075).

Swallows successfully produced at least one fledgling in 47.4% of 133 nests. We could identify causes of breeding failure in 12 cases. Eleven nests were depredated probably by mammals, whereas the remaining nest was abandoned by adults. Model selection of nesting success analyses of the first model set yielded several high ranked models that contained combinations of all the explanatory variables (Table 3). Model averaging results showed that distance to edge and habitat type, which were not included in the best model (Table 3), did not influence nesting success of swallows (Table 4). Conversely, ecosystem engineer identity was included in the best model and had a strong influence on nesting success (Tables 3 and 4). Swallows were less likely to produce at least one fledgling in flicker cavities than in sapsucker or hairy woodpecker cavities, whereas the probability of nesting success in cavities excavated by woodpeckers did not differ significantly from that in cavities supplied by fungal/insect decay (Figure 4).

**Table 3:** Model selection of analyses that examined nesting success of swallows and bluebirds.

Species	Model set	Models	df	LogLik	AICc	ΔAICc	Weight
**Swallow**	1	Stage, ecosystem engineer	9	-83.24	185.93	0.00	0.27
**Swallow**	1	Stage, ecosystem engineer, edge	10	-82.43	186.67	0.73	0.19
**Swallow**	1	Stage	6	-87.40	187.46	1.53	0.13
**Swallow**	1	Stage, ecosystem engineer, habitat	10	-82.85	187.50	1.57	0.12
**Swallow**	1	Stage, edge	7	-86.42	187.73	1.80	0.11
**Swallow**	1	Stage, ecosystem engineer, habitat, edge	11	-82.24	188.66	2.72	0.07
**Swallow**	1	Stage, habitat	7	-87.33	189.56	3.62	0.04
**Swallow**	1	Stage, habitat, edge	8	-86.41	189.99	4.06	0.04
**Swallow**	1	Intercept-only model	4	-90.88	190.08	4.15	0.03
**Swallow**	2	Stage, height	7	-83.75	182.40	0.00	0.22
**Swallow**	2	Stage, height, entrance	8	-82.67	182.50	0.11	0.21
**Swallow**	2	Stage, height, volume	8	-83.09	183.34	0.95	0.14
**Swallow**	2	Stage, height, habitat, edge	9	-82.35	184.16	1.76	0.09
**Swallow**	2	Stage, height, entrance, volume	9	-82.38	184.22	1.82	0.09
**Swallow**	2	Stage, entrance	7	-85.09	185.08	2.68	0.06
**Swallow**	2	Stage, entrance, volume	8	-84.16	185.48	3.08	0.05
**Swallow**	2	Stage, volume	7	-85.29	185.49	3.09	0.05
**Swallow**	2	Stage, height, entrance, volume, habitat, edge	11	-81.08	186.33	3.94	0.03
**Swallow**	2	Stage	6	-87.40	187.46	5.06	0.02
**Swallow**	2	Stage, edge	7	-86.42	187.73	5.33	0.02
**Swallow**	2	Stage, entrance, edge, habitat	9	-84.17	187.80	5.41	0.01
**Swallow**	2	Stage, edge, habitat, volume	9	-84.23	187.92	5.53	0.01
**Swallow**	2	Stage, habitat	7	-87.33	189.56	7.16	0.01
**Swallow**	2	Stage, habitat, edge	8	-86.41	189.99	7.59	0.00
**Swallow**	2	Intercept-only model	4	-90.88	190.08	7.68	0.00
**Bluebird**	1	Intercept-only model	4	-113.76	235.77	0.00	0.37
**Bluebird**	1	Stage, habitat	7	-110.65	236.01	0.25	0.33
**Bluebird**	1	Stage, habitat, edge	8	-110.43	237.77	2.00	0.14
**Bluebird**	1	Stage	6	-112.93	238.39	2.63	0.10
**Bluebird**	1	Stage, edge	7	-110.60	239.96	4.19	0.05
**Bluebird**	1	Stage, ecosystem engineer, habitat	10	-112.27	242.62	6.86	0.01
**Bluebird**	1	Stage, ecosystem engineer	9	-110.37	243.69	7.93	0.01
**Bluebird**	1	Stage, ecosystem engineer, habitat, edge	11	-112.06	244.45	8.69	0.00
**Bluebird**	1	Stage, ecosystem engineer, edge	10	-82.43	245.54	9.78	0.00
**Bluebird**	2	Intercept-only model	4	-113.76	235.77	0.00	0.31
**Bluebird**	2	Stage, habitat	7	-110.65	236.01	0.25	0.27
**Bluebird**	2	Stage, habitat, edge	8	-110.43	237.77	2.00	0.11
**Bluebird**	2	Stage	6	-112.93	238.39	2.63	0.08
**Bluebird**	2	Stage, height	7	-112.36	239.42	3.66	0.05
**Bluebird**	2	Stage, entrance	7	-112.50	239.71	3.94	0.04
**Bluebird**	2	Stage, entrance, edge, habitat	9	-110.33	239.81	4.04	0.04
**Bluebird**	2	Stage, edge	7	-112.62	239.96	4.19	0.04
**Bluebird**	2	Stage, height, habitat, edge	9	-110.41	239.98	4.22	0.04
**Bluebird**	2	Stage, height, entrance	8	-112.11	241.15	5.38	0.02
**Bluebird**	2	Stage, volume	7	-116.28	247.26	11.50	0.00
**Bluebird**	2	Stage, volume, entrance	8	-115.47	247.87	12.10	0.00
**Bluebird**	2	Stage, edge, habitat, volume	9	-114.50	248.16	12.40	0.00
**Bluebird**	2	Stage, volume, height	8	-115.77	248.45	12.69	0.00
**Bluebird**	2	Stage, height, entrance, volume	9	-115.23	249.62	13.85	0.00
**Bluebird**	2	Stage, height, entrance, volume, habitat, edge	11	-114.11	251.94	16.17	0.00

Model set 1 included the stage at which nests were found, distance from cavities to forest edge, ecosystem engineer identity and habitat type (aspen groves vs. continuous forest) as fixed terms. For model set 2, cavity dimension variables (cavity entrance size, volume and height above the ground level) were fitted as fixed terms instead of ecosystem engineer identity. The rest as in table 1.

**Table 4:** Model-averaged parameter estimates and standard errors for analyses that examined nesting success of swallows.

Model set	Parameters	Estimate ± SE	*z*	*p*
**1**	Intercept	0.841 ± 1.430	0.588	0.557
**1**	Stage (egg laying/incubation)	-1.392 ± 1.325	1.051	0.293
**1**	Stage (pre-laying)	-0.251 ± 1.334	0.188	0.851
**1**	Ecosystem engineer (rot-fungi/insects)	-0.368 ± 0.876	0.421	0.674
**1**	Ecosystem engineer (flickers)	-1.465 ± 0.617	2.374	**0.018**
**1**	Ecosystem engineer (sapsuckers)	-0.277 ± 0.603	0.459	0.646
**1**	Distance to edge	0.015 ± 0.015	1.000	0.317
**1**	Habitat (continuous forest)	-0.402 ± 0.624	0.644	0.520
**2**	Intercept	0.104 ± 1.844	0.056	0.955
**2**	Stage (egg laying/incubation)	-1.718 ± 1.329	1.292	0.196
**2**	Stage (pre-laying)	-0.436 ± 1.336	0.326	0.744
**2**	Cavity height	0.480 ± 0.211	2.269	**0.023**
**2**	Cavity entrance size	-0.249 ± 0.168	1.480	0.139
**2**	Cavity volume	-0.0001 ± 0.0001	1.102	0.270
**2**	Distance to edge	0.014 ± 0.014	0.971	0.331
**2**	Habitat (continuous forest)	-0.533 ± 0.681	0.783	0.433

Significant values (*p*≤0.05) are highlighted in bold.

Similar to previous analyses, results of the second model set show that distance to edge and habitat type did not have a strong influence on nesting success for tree swallows (Tables 3 and 4). In addition, cavity entrance size and cavity volume were not significantly associated with nesting success probability (Table 4). However, swallow nests in cavities located higher on trees were more successful than nests in lower cavities (mean cavity height ± SE: successful vs. failed nesting cavities = 3.2 ± 0.2 m vs. 2.8 ± 0.1 m, Table 4). Because nesting success analyses were based on a subsample of accessible cavities (up to 5.2 m, see methods section), we ran another model with a larger data set that considered the whole variation in cavity heights. In accordance with previous analyses, nests in higher cavities were more successful than nests in lower cavities (mean cavity height ± SE: successful vs. failed nesting cavities = 5.0 ± 0.2 m vs. 4.1 ± 0.2 m, parameter estimate ± SE = 0.120 ± 0.055, *z*=2.158, *p*=0.03, n=320 nests).

Bluebirds successfully produced at least one fledgling in 55.7% of 167 nests. We could identify causes of breeding failure in 21 cases. Twelve nests (57.1%) were depredated probably by mammals. An adult was found depredated at the base of the cavity-tree in two cases, whereas nest abandonment or starvation led to nest losses in the remaining 7 cases. The intercept-only model was the highest ranked model in both model sets that examined nesting success of bluebirds (Table 3), indicating that none of the predictors had a strong influence.

### Clutch size and fledgling number of secondary cavity users

Clutch size was examined in 45 and 111 nests of swallows (mean = 5.02 eggs ± 0.19 SE) and bluebirds (5.17 ± 0.07), respectively. Analyses of the variation in clutch size show that the intercept-only model was the highest ranked model in both model sets for each cavity nester (Table 5), indicating that predictors did not have a strong influence on clutch size.

**Table 5:** Model selection of analyses that examined clutch size of swallows and bluebirds.

Species	Model set	Models	df	LogLik	AICc	ΔAICc	Weight
**Swallow**	1	Intercept-only model	4	-7.64	24.29	0.00	0.56
**Swallow**	1	Habitat	5	-7.48	26.49	2.21	0.19
**Swallow**	1	Edge	5	-7.53	26.60	2.31	0.18
**Swallow**	1	Habitat, edge	6	-7.44	29.10	4.81	0.05
**Swallow**	1	Ecosystem engineer	7	-7.07	31.16	6.88	0.02
**Swallow**	1	Ecosystem engineer, edge	8	-6.90	33.80	9.51	0.00
**Swallow**	1	Ecosystem engineer, habitat	8	-7.01	34.02	9.73	0.00
**Swallow**	1	Ecosystem engineer, habitat, edge	9	-6.90	36.93	12.65	0.00
**Swallow**	2	Intercept-only model	4	-7.64	24.29	0.00	0.58
**Swallow**	2	Habitat	5	-7.48	26.49	2.21	0.19
**Swallow**	2	Edge	5	-7.53	26.60	2.31	0.18
**Swallow**	2	Habitat, edge	6	-7.44	29.10	4.81	0.05
**Swallow**	2	Volume	5	-54.14	119.81	95.53	0.00
**Swallow**	2	Volume, edge	6	-54.12	122.45	98.16	0.00
**Swallow**	2	Volume, habitat	6	-54.13	122.48	98.19	0.00
**Swallow**	2	Volume, habitat, edge	7	-54.12	125.27	100.98	0.00
**Bluebird**	1	Intercept-only model	5	-124.85	260.28	0.00	0.42
**Bluebird**	1	Edge	6	-124.52	261.85	1.57	0.19
**Bluebird**	1	Habitat	6	-124.64	262.08	1.80	0.17
**Bluebird**	1	Habitat, edge	7	-124.32	263.72	3.44	0.07
**Bluebird**	1	Ecosystem engineer	8	-123.23	263.88	3.60	0.07
**Bluebird**	1	Ecosystem engineer, edge	9	-122.54	264.87	4.59	0.04
**Bluebird**	1	Ecosystem engineer, habitat	9	-123.14	266.05	5.78	0.02
**Bluebird**	1	Ecosystem engineer, habitat, edge	10	-122.33	266.87	6.59	0.02
**Bluebird**	2	Intercept-only model	5	-124.85	260.28	0.00	0.36
**Bluebird**	2	Edge	6	-124.52	261.85	1.57	0.17
**Bluebird**	2	Habitat	6	-124.64	262.08	1.80	0.15
**Bluebird**	2	Volume	6	-124.79	262.40	2.12	0.13
**Bluebird**	2	Habitat, edge	7	-124.32	263.72	3.44	0.06
**Bluebird**	2	Volume, edge	7	-124.42	263.93	3.66	0.06
**Bluebird**	2	Volume, habitat	7	-124.55	264.19	3.92	0.05
**Bluebird**	2	Volume, habitat, edge	8	-124.19	265.79	5.52	0.02

Model sets 1 included the distance from cavities to forest edge, ecosystem engineer identity and habitat type (aspen groves vs. continuous forest) as fixed terms. For model sets 2, cavity volume was fitted as a fixed term instead of ecosystem engineer identity. The rest as in table 1.

The number of fledglings of successful broods was examined in 59 and 89 nests of swallows (mean = 3.81 fledglings ± 0.18 SE) and bluebirds (4.13 ± 0.12), respectively. Similar to clutch size analyses, the intercept-only model was the highest ranked model in both model sets that assessed the variation in the number of fledglings for each species (Table 6), again suggesting low influence of explanatory variables.

**Table 6:** Model selection of analyses that examined the number of fledglings of swallows and bluebirds.

Species	Model set	Models	df	LogLik	AICc	ΔAICc	Weight
**Swallow**	1	Intercept-only model	4	-15.58	39.90	0.00	0.56
**Swallow**	1	Habitat	5	-15.55	42.24	2.34	0.17
**Swallow**	1	Edge	5	-15.58	42.29	2.39	0.17
**Swallow**	1	Habitat, edge	6	-15.55	44.72	4.83	0.05
**Swallow**	1	Ecosystem engineer	7	-14.67	45.53	5.64	0.03
**Swallow**	1	Ecosystem engineer, habitat	8	-14.64	48.16	8.26	0.01
**Swallow**	1	Ecosystem engineer, edge	8	-14.65	48.18	8.28	0.01
**Swallow**	1	Ecosystem engineer, habitat, edge	9	-14.63	50.92	11.03	0.00
**Swallow**	2	Intercept-only model	4	-15.58	39.90	0.00	0.59
**Swallow**	2	Habitat	5	-15.55	42.24	2.34	0.18
**Swallow**	2	Edge	5	-15.58	42.29	2.39	0.18
**Swallow**	2	Habitat, edge	6	-15.55	44.72	4.83	0.05
**Swallow**	2	Volume	5	-64.00	139.13	99.23	0.00
**Swallow**	2	Volume, edge	6	-64.00	141.62	101.72	0.00
**Swallow**	2	Volume, habitat	6	-64.02	141.65	101.75	0.00
**Swallow**	2	Volume, habitat, edge	7	-64.02	144.23	104.33	0.00
**Bluebird**	1	Intercept-only model	4	-14.60	37.67	0.00	0.55
**Bluebird**	1	Habitat	5	-14.58	39.87	2.20	0.18
**Bluebird**	1	Edge	5	-14.58	39.89	2.22	0.18
**Bluebird**	1	Habitat, edge	6	-14.56	42.15	4.48	0.06
**Bluebird**	1	Ecosystem engineer	7	-14.50	44.38	6.71	0.02
**Bluebird**	1	Ecosystem engineer, edge	8	-14.48	46.76	9.09	0.01
**Bluebird**	1	Ecosystem engineer, habitat	8	-14.49	46.78	9.11	0.01
**Bluebird**	1	Ecosystem engineer, habitat, edge	9	-14.47	49.23	11.56	0.00
**Bluebird**	2	Intercept-only model	4	-14.60	37.67	0.00	0.57
**Bluebird**	2	Habitat	5	-14.58	39.87	2.20	0.19
**Bluebird**	2	Edge	5	-14.58	39.89	2.22	0.19
**Bluebird**	2	Habitat, edge	6	-14.56	42.15	4.48	0.06
**Bluebird**	2	Volume	5	-85.71	182.14	144.47	0.00
**Bluebird**	2	Volume, habitat	6	-85.71	184.44	146.77	0.00
**Bluebird**	2	Volume, edge	6	-85.72	184.47	146.79	0.00
**Bluebird**	2	Volume, habitat, edge	7	-85.72	186.82	149.14	0.00

Instead of ecosystem engineer identity, cavity volume was fitted as a fixed term in the second model sets. The rest as in table 1.

## Discussion

We investigated the mechanisms underlying the specific associations between ecosystem engineers and secondary cavity users in an avian nest web community. For this purpose, we examined the response of two coexisting cavity-nesting songbirds with strong overlapping habitat requirements to changes in the quantity and quality of resources (i.e. nesting cavities) supplied by ecosystem engineers. The most abundant tree cavities supplied by red-naped sapsuckers (52% of cavities, see also Figure 1) had low probabilities of being used by mountain bluebirds compared to other cavity types. Likewise, tree swallows were less likely to use northern flicker cavities than other cavity types, despite the high abundance of flicker cavities (33% of cavities) compared to cavities supplied by hairy woodpeckers and fungal/insect decay (Figure 1). In addition, swallows were less likely to breed successfully in flicker cavities, suggesting that flickers provide swallows with lower quality cavities than the other excavators. Thus, resource quality can be an important feature to understanding the inter-specific associations between ecosystem engineers and resource users. However, most studies that examine the outcome of ecosystem engineering have not considered the quality of species-specific resources for each user provided by particular ecosystem engineer species. Given the extensive relevance of ecosystem engineering as a structuring force in ecological communities [2,3], we urge the incorporation of resource quality parameters into models that assess community dynamics to achieve a better understanding of ecological communities.

Theory predicts that niche differentiation may promote coexistence of ecologically similar species by overcoming potential negative effects of inter-specific competition [13,14]. The differences in tree cavity use and reproductive performance of secondary cavity nesters in different cavity types may explain, at least partly, niche differentiation and coexistence of our ecologically similar songbird species (swallows and bluebirds), which share foraging and nesting habitats but exhibit contrasting probabilities of use and reproductive success in flicker and sapsucker cavities.

An appropriate understanding of community dynamics requires a proper knowledge of the outcome of associations between ecosystem engineers and resource users [6]. Our results indicate low reproductive outputs of swallows in flicker cavities compared to those in sapsucker cavities (1.1 vs. 2.1 fledglings per nest), showing that, even if flicker cavities were used extensively (30.9% of swallow nests in flicker cavities), the use of flicker cavities may reduce swallow fitness. Instead, swallows greatly benefit by nesting in cavities excavated by sapsuckers, which provide swallows with numerous (41.1%) high quality nest-sites. This may explain results from previous studies showing that tree swallows mostly rely on sapsuckers to produce their nest cavities [32] and, in consequence, are restricted to sites occupied by sapsuckers [33]. On the other hand, bluebird populations largely benefit from using flicker cavities, as flickers provide numerous (57.7%) suitable nest-sites for bluebirds. Thus, the identification of key ecosystem engineers may require the evaluation of resource quality based on the assessment of the factors that influence resource use and reproduction for each resource user.

Our results also suggest that some ecosystem engineers may be functionally similar for swallows and bluebirds. While swallows appeared to prefer the less common hairy woodpecker cavities even more than the abundant and frequently-used sapsucker cavities, the reproductive parameters of swallows were similar in the cavities of both ecosystem engineers, suggesting that the quality of hairy woodpecker cavities was at least as high as that of sapsucker cavities. Hairy woodpecker cavities were as likely to be used by bluebirds as flicker cavities, which were highly used by bluebirds, and bluebirds exhibited comparable reproductive output in both types of cavities. This suggests that the low use of hairy woodpecker cavities by swallows (13.1% of nests in hairy woodpecker cavities) and bluebirds (13.3%) was associated with the low quantity of hairy woodpecker cavities (9% of cavities) compared to other types of cavities (Figure 1) rather a low quality of hairy woodpecker cavities.

Most nest losses of swallows were due to nest predation by mammals in flicker cavities, which were located low on the trees compared to other cavity types. Swallow nesting success was not related to other habitat or cavity attributes apart from the identity of the ecosystem engineer and cavity height above the ground level. The high predation in cavities located low on the trees matches the results from Fisher and Wiebe [29] showing that flicker nests located lower on the trees were subjected to higher mammalian predation in an area that partly overlaps our study area.

We suggest that higher nest losses of swallows (but not of bluebirds) in flicker cavities compared to other cavity types may be associated with an increase in predation risk of these cavities during the peak of the swallow breeding season. Indeed, several studies show that the risk of nest predation varies during the breeding season so that timing of breeding of birds has been suggested as an important determinant for nest predation risk [25,29,34]. Contrary to swallows, nesting success of bluebirds did not differ significantly between flicker cavities and other cavity types, perhaps because bluebirds nest earlier in the season [24] and thus may escape the peak of nest predation in flicker cavities. Other factors may also explain the low breeding success of swallows (but not of bluebirds) associated with elevated predation risk in flicker cavities. Bluebirds might be more effective in deterring predator attacks or less conspicuous to predators than swallows. If so, bluebirds might be better able to cope with the potentially increased predation risk than swallows. Another possibility is that flicker cavities are mostly occupied by young or inexperienced swallows that are less able to deter predator attacks or that behave more conspicuously when predators are present, which may make them relatively more vulnerable to predators, whereas low proportions of young/inexperienced bluebirds might occupy flicker cavities. Unfortunately, we lack the data with banded birds to examine any of these alternatives.

Bluebirds were less likely to nest in sapsucker cavities than in other cavity types. In accordance with this result, only 12% of bluebird nests were located in sapsucker cavities despite the high relative abundance of these cavities (52% of cavities, see also Figure 1). Even if sapsuckers are on average slightly larger than bluebirds, bluebirds preferred to occupy sapsucker cavities with larger entrances, which may suggest that the small entrance size of many sapsucker cavities may prevent the access to bluebirds. Indeed, Haeckler [35] suggested that bluebirds did not enter nest-boxes with an entrance size of 3.8 cm, but they did use these boxes after the entrance enlarged to 4.4 cm, which matches our results stating that bluebirds used sapsucker cavities with an average entrance size of 4.3 cm and did not use cavities with an average entrance size of 3.9 cm. However, if bluebirds could not enter small sapsucker cavities or if they preferred larger cavities for another unknown reason remains unclear and deserves more research. Other structural factors do not seem to explain the low probability of using sapsucker cavities compared to other cavity types, as we did not find significant differences in cavity volume, cavity height on the trees or cavity distances to forest edge between sapsucker and hairy woodpecker cavities; the latter cavities having higher probability of being occupied by bluebirds (Figure 3). Moreover, we found no effect of cavity type on bluebird nesting success, clutch size or number of fledglings, nor an effect of cavity entrance size on bluebird nesting success, which may suggest that the low use of small sapsucker cavities was not associated with reduced fecundity for bluebirds nesting in these cavities.

In North America, avian excavators provide most nesting sites for secondary cavity nesters, whereas cavities created by fungal or insect decay appear to be less important (e.g. [17,36–39]). We suggest that the low importance of cavities supplied by fungal/insect decay may be associated with their low abundance (6% of cavities, see also Figure 1) but not with a low quality of these cavities per se, as reproductive performance of both swallows and bluebirds did not differ significantly between cavities supplied by fungal/insect decay and cavities supplied by the key ecosystem engineers for each secondary cavity nester (i.e. sapsuckers for swallows and flickers for bluebirds).

Cavities in aspen groves and close to forest edges were more likely to be used by both swallows and bluebirds. These patterns may be explained by the high availability of open areas surrounding aspen groves and the proximity of open habitats close to forest edges, as open habitats (grasslands and shallow ponds) provide swallows and bluebirds with suitable foraging habitat [22,23]. However, nesting success of both cavity nesters did not vary with habitat type or proximity to forest edges, which may suggest that nest predation pressure was not higher in continuous forests or the forest interior. In addition, clutch size and fledgling numbers, two reproductive parameters associated with food supply during pre-breeding and breeding seasons, were not higher in aspen groves or close to open areas. Thus, we hypothesize that a potential increase in foraging opportunities in aspen groves may affect the occurrence and abundance, but not the fecundity, of swallows and bluebirds. However, other potential factors not examined in this study, such as an increased predation pressure on adults in continuous forests compared to aspen groves may also explain the preference of bluebirds and swallows for aspen groves.

Our results can inform conservation efforts aimed at ecological communities based on ecosystem engineering. Because ecosystem engineers may provide resources that differ in quality for each resource user, the conservation of populations of resource users may require the development of different management actions for each key ecosystem engineer. In our study, an increase in the abundance of cavities supplied by flickers would benefit mostly bluebirds, whereas an increase of sapsucker cavities would impact positively mainly swallow populations. This is particularly relevant because swallow populations have declined in large areas in North America during recent decades [40]. While high abundance of recently dead aspen trees may provide suitable substrate for excavation of flicker cavities, live aspen with external signs of decay provide the most suitable excavation substrate for sapsuckers [41]. Live unhealthy aspen also provide suitable excavation substrate for hairy woodpeckers [41], which provide high quality nest-sites for bluebirds and swallows. In addition, although cavities supplied by fungal/insect decay have been suggested to hold low importance for the entire guild of secondary cavity nesters on the basis of low cavity use [39], these cavities provide swallows and bluebirds with suitable nest-sites that deserve protection.
